# Long-Term Outcome of Treating Periprosthetic Hip Joint Infection with Local Antibiotics Delivered Through Antibiotic-Impregnated Calcium Hydroxyapatite

**DOI:** 10.3390/jcm13237469

**Published:** 2024-12-08

**Authors:** Hiroki Wakabayashi, Masahiro Hasegawa, Yohei Naito, Shine Tone, Akihiro Sudo

**Affiliations:** Department of Orthopaedic Surgery, Mie University Graduate School of Medicine, 2-174 Edobashi, Tsu 514-8507, Japan; masahase@clin.medic.mie-u.ac.jp (M.H.); yo-yo@clin.medic.mie-u.ac.jp (Y.N.); s-tone@clin.medic.mie-u.ac.jp (S.T.); a-sudou@clin.medic.mie-u.ac.jp (A.S.)

**Keywords:** hip joint, infection, prosthesis, long-term, outcome, antibiotic, calcium hydroxyapatite

## Abstract

**Background/Objectives**: This study explores the long-term clinical outcomes of antibiotic-impregnated calcium hydroxyapatite (CHA) as an antibiotic delivery system in treating periprosthetic joint infection (PJI) following total hip arthroplasty (THA). **Methods**: We conducted a retrospective analysis of 12 patients (13 hips) who were treated with antibiotic-impregnated CHA for PJI after THA and followed for more than 10 years at our institution between 1999 and 2011. The study group comprised six men (seven hips) and six women, with a mean age of 61.4 years. **Results**: The mean follow-up duration was 13.8 years. After irrigation and debridement with modular component exchange, seven hips in six patients underwent revision surgery; however, PJI relapsed in two hips of two patients with a history of diabetes. Two-stage revision surgery was performed on the two relapsed hips and six scheduled hips with antibiotic-impregnated CHA used to treat all cases of PJI. Infection control (100% rate) was achieved in all joints, and revision surgeries were completed. Two patients died 12 years after the initial procedure, and one died 14 years after the first procedure due to unrelated internal diseases; no infection recurrence was observed. No complications related to antibiotic-impregnated CHA were observed. **Conclusions**: Our results indicate that antibiotic-impregnated CHA is associated with high success rates in treating PJI after THA, even in cases with advanced disease, and yields satisfactory functional outcomes postoperatively.

## 1. Introduction

Periprosthetic joint infection (PJI) is a serious complication that can occur after joint replacement surgery [1]. Large-scale registry studies indicate that the incidence of PJI is around 1% for total hip arthroplasty (THA) and 2% for total knee arthroplasty (TKA) [2,3]. PJI in the hip joint greatly diminishes patients’ quality of life [2,4]. It significantly influences morbidity and mortality and is usually managed through a combination of surgical procedures and antibiotic treatment. Long-term administration of oral antibiotics after surgery is frequently recommended for managing PJI [5].

PJI remains one of the difficult treatment complications of total joint arthroplasty (TJA), with two-stage revision considered the gold-standard treatment [6,7]. Recently, one-stage revision surgery has gained popularity and has been reported to achieve infection eradication rates comparable to those of two-stage revision surgery in appropriately selected patients (67–100%). However, certain patients may not be suitable for this approach [8]. Various factors, such as the presence of fistula or bone loss, high antibiotic resistance, unknown infecting microorganisms, poor host status, or prior failure to eradicate the infection, can hinder the use of one-stage revision surgery [9]. Therefore, two-stage revision remains the preferred standard. Since the probability of eradicating an infection after a failed initial two-stage revision is low [10], reducing the incidence of reinfection is crucial [11].

The application of local antibiotics in combination with carrier materials during PJI revision surgery and THA revision surgery has the potential to enhance infection-free survival rates. In spinal surgery, local administration of vancomycin powder (VP) has been demonstrated to effectively and safely reduce infection rates [12]. Research in the field of spinal surgery has shown that applying local vancomycin before wound closure significantly lowers the incidence of surgical site infections [12]. Regarding PJI treatment, Martin et al. reported that the infection rate in the control group was 3.6%, whereas it markedly dropped to 1.43% with the use of VP at the surgical site [13].

Debridement with implant retention, recently approved for the treatment of PJI, is an attractive treatment option for early PJI as it can reduce morbidity, hospitalization duration, and healthcare costs compared to one-stage or two-stage revision arthroplasty.

**Figure 1:**
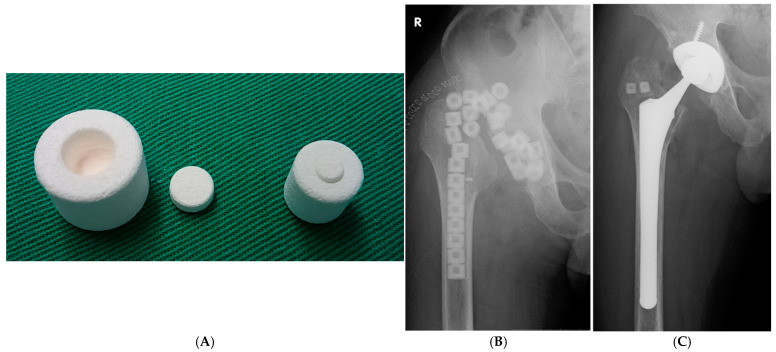
(**A**): Photograph of a calcium hydroxyapatite (CHA) ceramic block. (**B**): Radiographs of the right hip of a 58-year-old man (Case 9). Anteroposterior radiograph after the removal of all components and insertion of antibiotic-impregnated CHA into the bone and joint space. (**C**): Anteroposterior X-ray image after revision total hip arthroplasty, showing CHA blocks implanted in the acetabulum and major trochanter.

Antibiotic-impregnated calcium hydroxyapatite (CHA) ceramic has recently been developed as a drug delivery system (Figure 1A) [14,15], and it has been used in the treatment of PJI after THA. This system enables the complete release of implanted antibiotics over an extended period without drug entrapment in the composite [14], demonstrating high release rates [14]. We have employed this drug delivery system with CHA to treat patients with PJI. However, few studies have reported the long-term outcomes of PJI treatment. The long-term results of I&D with modular component exchange or two-stage revision procedures and infection-free prosthesis survival in a single-center remain unknown. Therefore, we aimed to assess the long-term clinical outcomes of antibiotic-impregnated CHA in the treatment of hip PJIs.

## 2. Materials and Methods

This study was approved by our Institutional Review Board. Twelve patients (13 hips) treated for PJI after THA at our institution between 1999 and 2011 were retrospectively enrolled. All patients had a follow-up period of more than 10 years after treatment. The study group included six men (seven hips) and six women, with a mean age of 61.4 years (range: 35–71 years). The initial diagnoses included osteoarthritis in six patients, rheumatoid arthritis in one patient (both hips), and femoral neck fracture in five patients (Table 1).

The diagnosis of infection was based on clinical criteria, including the presence of a discharging sinus, purulent fluid, or pus found during preoperative hip aspiration, or positive laboratory and histopathological findings. PJI was defined as the presence of a sinus tract communicating with the prosthesis, at least two identical positive cultures, or both.

The soft tissue was normal in 11 hips (ten patients), while two hips had abscesses and fistulas (Table 2). One of the two hips with a fistula developed a productive fistula 4 days preoperatively (case 4).

Surgery

Irrigation and debridement (I&D)

Open debridement was performed using the previous incision and approach [16]. Extensive synovectomy of all joint synovial tissues affected by abscess and necrosis around the prosthetic joint was performed, followed by thorough lavage with antibiotic irrigation. In the I&D protocol with modular component exchange, the modular components (including the femoral head and acetabular insert) and any loose components were replaced, while the fixed components were retained [16]. Six patients (seven hips) underwent I&D with modular component exchange (Table 3). In case 12, due to cup loosening, the cup was also revised.

Treatment of antibiotic-impregnated CHA

All patients underwent I&D with modular component exchange, reimplantation with a two-stage revision using antibiotic-impregnated CHA applied to the major trochanter, acetabulum, or both (Figure 1B). CHA (Bone Ceram P; Olympus Terumo Biomaterials Corp, Tokyo, Japan) in cylindrical shapes with 30–40% porosity and micropore diameters between 40 and 150 µm was used. Two sizes of cylindrical blocks (large and small) were employed.

In the two-stage revision, after all components were removed, a large block (15 mm in diameter and 12 mm in height) with a central cylindrical cavity was used. Antibiotic-impregnated CHA was inserted into the bone and joint spaces. Each ceramic block contained 100–400 mg of antibiotics, based on bacterial culture results from the preoperative joint aspirations (Table 4).

For I&D and two-stage reimplantation, a smaller stent (10 mm in diameter and 10 mm in height) was used. Intraoperatively, the sensitive selected antibiotic powder was packed into the central cylindrical cavity of each porous block. Typical amounts of antibiotics were placed in each ceramic block. The details of antibiotics used to impregnate the CHA are listed in Table 2 and Table 3. In six patients (seven hips), vancomycin hydrochloride (VCM) was used for CHA impregnation. Bone holes were created in the acetabulum and greater trochanter using an air drill. We applied these CHA blocks in the major trochanter, the acetabulum, and into the femoral canal during revision surgery. We implanted CHA blocks impregnated with antibiotics as frequently as possible in areas where bone mass remained. We selected the antibiotics to be packed into the CHA blocks based on the sensitivity of the wound pathogens. Typically, 2–5 CHA blocks were implanted into the bone holes. In cases 6 and 7, CHA was not implanted due to severe bone loss.

Two-stage revision protocol

In the two-stage revision surgery protocol explained in the previous report [17], all components were removed in the first stage, followed by meticulous debridement, and antibiotic-laden CHA was inserted into the bone and joint cavity. Depending on the extent of the bone defect, 20 to 30 large CHA blocks were utilized in each case (Figure 1B).

The timing of the second-stage revision surgery (reimplantation) was determined based on infection control and clinical symptoms. The criteria for THA reimplantation included wound healing, normal C-reactive protein (CRP) levels, and negative culture results after component removal.

Antibiotic therapy

Antibiotic therapy was initiated after preoperative hip aspiration. Intravenous (IV) antibiotics were administered at an effective dose and adjusted based on the aspiration findings. Antibiotic-impregnated CHA served as local antibiotic therapy, while IV antibiotic therapy was maintained for 3–9 weeks, followed by oral antibiotics for 6 weeks to 4 months. Patient tolerance to this regimen was satisfactory.

Evaluation of outcomes

The primary outcome measure was the presence or absence of PJI at the most recent clinical follow-up or on the final follow-up date. This assessment included blood tests, such as erythrocyte sedimentation rate and CRP levels.

Clinical hip function outcomes were evaluated using the Japanese Orthopaedic Association (JOA) hip score, which has a maximum score of 100 points, representing no disability [18].

Success criterion

Treatment success was defined as the absence of infection after 2 years of prosthesis retention, as described previously [16,19].

Statistical Analysis

Statistical analyses were performed using the non-parametric Wilcoxon signed-rank test, analysis of variance, and Spearman’s rank correlation coefficient. Statistical significance was set at *p* < 0.05. All statistical analyses were performed using the IBM SPSS Statistics software26 (IBM Japan, Tokyo, Japan).

## 3. Results

Pre-operative patient information

All patients underwent preoperative hip aspiration and standard microbiological (aerobic and anaerobic) cultures. In 13 aspirates, the causative bacterium was identified in all but two samples. In nine patients (10 hips), Staphylococcus aureus was the causative microorganism, including 1 hip with methicillin-resistant S. aureus (MRSA). In two patients, the Streptococcus sp. was identified as the causative organism. One patient had a fungal infection caused by Candida glabrata, and in one patient, the causative microorganism remained unknown (cases 11 and 12) (Table 2). The patient without bacterial identification showed purulent fluid and pus during preoperative hip aspiration and intraoperative evaluation. The diagnosis of PJI was confirmed based on clinical, intraoperative macroscopic, and histological findings.

Only two patients (three hips, cases 4 and 5) presented with infectious symptoms for less than 3 weeks, while the other patients had symptoms lasting for more than 1 month.

All patients (all hips) were successfully treated and had no signs of infection at the latest follow-up. Three hips in which primary treatment failed (cases 2, 3, and 8) were successfully treated with two-stage reimplantation and antibiotic-impregnated CHA. In one hip (case 8), persistent infection was suspected at 3 months and 1-year post-removal, due to the coexistence of MRSA and Candida glabrata. Re-debridement with CHA exchange was performed before reimplantation (Table 4, case 8). Except for case 8, which required intravenous (IV) antibiotic administration for 10 weeks to achieve a normal CRP level, the other patient’s CRP levels normalized in 2–3 weeks postoperatively with IV antibiotics (Table 3 and Table 4).

The three patients who failed primary treatment (cases 2, 3, and 8) had diabetes and infectious symptoms for more than 1 month. No postoperative complications, such as excessive drainage, erythema, bone damage due to friction, or particulate disease, were observed in any of the patients after treatment with antibiotic-impregnated CHA.

Treatment outcomes

The mean follow-up duration was 13.8 years (range: 11–19.7 years). Implant-related complications included one joint dislocation 3 days postoperatively and cup loosening 10 years postoperatively. At the final evaluation, no implant loosening was observed, although 10 joints exhibited a stress shielding degree of three or higher (Table 5).

Two patients died 12 years after the first surgery, and one patient died 14 years after the first surgery due to unrelated internal diseases. However, there was no recurrence of infection in any of the patients.

Functional outcomes

At the most recent follow-up, the mean JOA hip score was 69.3 (range: 46–95). Although the difference was not statistically significant, the mean JOA score was 73.8 points for the five hips in which the implants were preserved, while the eight hips that underwent two-stage revision surgery (including the two relapsed hips and six scheduled hips) had a lower mean score of 66.5 points, but there was no significant difference.

Case 9. A 58-year-old man

A fifty-eight year-old patient sustained a right femoral neck fracture due to trauma and underwent right hemiarthroplasty at a nearby hospital. Four weeks after the surgery, pain and fever appeared. MSSA (methicillin-susceptible Staphylococcus aureus) was detected through aspiration and culture. Six months after the initial replacement, debridement and implant removal were performed, and antibiotic-impregnated CHA was inserted into the bone and joint space (Figure 1B).

**Figure 2:**
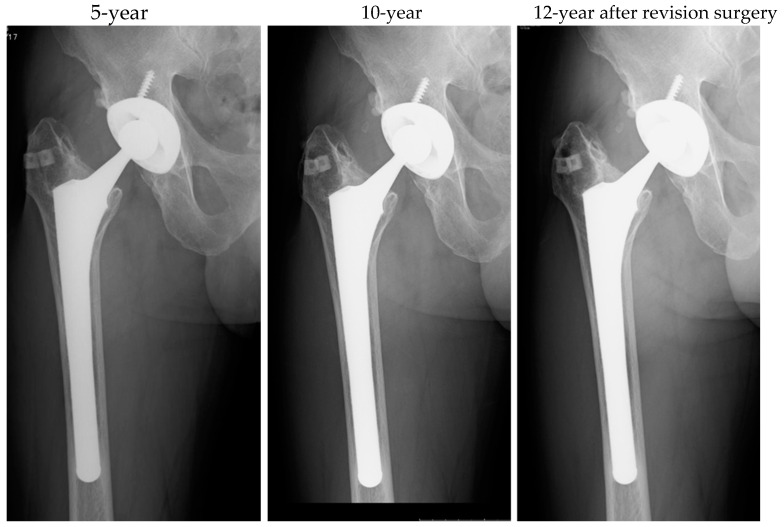
Anteroposterior radiograph after revision surgery in case 9.

Antibiotics were administered for three weeks, during which the CRP levels normalized. After stopping the antibiotics, CRP elevation was not observed. Seventy-eight days later, a reimplantation procedure was performed, with antibiotic-loaded HA embedded in the greater trochanter (Figure 1C). Up to 12 years after the final surgery, no reinfection occurred, and the implant remained stable with no loosening (Figure 2). The clinical course was favorable, and the JOA score at the 12-year mark was 95 points.

## 4. Discussion

In this study, we utilized antibiotic-impregnated CHA as a temporary antibiotic spacer instead of ALAC. Previous studies have shown that antibiotic-impregnated CHA releases antibiotics such as gentamicin sulfate, cefoperazone sodium, and flomoxef sodium for a longer duration compared to ALAC in both in vivo and in vitro settings [14]. Additionally, recent in vitro research demonstrated that antibiotic-impregnated CHA releases vancomycin hydrochloride (VCM) in higher concentrations and for a longer period compared to ALAC [20]. The antibiotics were packed in sufficient quantities within the block, so we believe that there was no uneven distribution. Therefore, we believe that the antibiotics studied showed a high release rate from the CHA block. And the advantage of CHA is that any antibiotic can be packed into the CHA because there is no damage to the drug due to polymerization heat.

With antibiotic-impregnated CHA as a temporary antibiotic spacer, the infection subsided with only one I&D, except in case 8, which was infected with MRSA and fungi. Polymicrobial and drug resistant-organism infections are challenging to treat and have high reinfection rates. In case 8, I&D was performed three times, but the infections were eventually cleared.

Although effective in eradicating infection, two-stage revision procedures are associated with worse functional outcomes [4,21,22], higher complication rates, and increased mortality compared to implant-retaining procedures or one-stage revision procedures [6].

A study from the Danish Registry reported a 14.6% reinfection rate at 5 years after reimplantation and an overall survival rate of 68% [23]. Similarly, Lange et al. conducted a systematic review and meta-analysis, estimating a reinfection rate of 10.4% (95% CI: 8.5–12.7%) following a two-stage revision [24]. A retrospective review by a single surgeon of 155 hips reported an overall survival rate of 91.7%, with a mean follow-up of 9.7 years and a mortality rate of 16.1% [25].

In terms of functional outcomes, a systematic review by Leonard et al. indicated that one-stage revision surgery yields better results [26]. The use of implant-retaining procedures and one-stage revision is gaining support. In carefully selected patients, the reinfection rate in one-stage revision is comparable to that in two-stage revision, but it appears to lead to better functional outcomes. I&D with modular component exchange offers a less invasive treatment option for early PJI, preserving bone stock, reducing operative time, lowering intraoperative fracture risk, and facilitating faster postoperative rehabilitation [16].

The development and maturation of biofilms over time in chronic PJI decrease treatment success rates [27]. However, high concentrations of antibiotics delivered locally may overcome and eradicate mature biofilms, contributing to the success of I&D in early PJI cases [28,29].

A recent study comparing debridement, antibiotics, and implant retention (DAIR) with or without local antibiotic delivery using calcium sulfate (CaSO4) as the carrier material showed that midterm success rates were lower without local antibiotic application [30]. In contrast, DAIR using antibiotic-loaded CaSO4 demonstrated reliable outcomes, with Kaplan–Meier analysis showing significantly longer infection-free survival when local antibiotics were used. This suggests that the success rate of DAIR may increase with local antibiotic delivery, especially when high concentrations of antibiotics cannot be applied directly around the prosthetic joint, as in cementless prostheses. It is suggested that this antibiotic delivery system could be a valuable tool for PJI surgeries using cementless prostheses. Furthermore, calcium hydroxyapatite ceramics, being entirely biocompatible, do not require complete removal during the second surgery.

Previous studies, including ours, have shown that two-stage revision procedures using CHA and I&D with modular component exchange can successfully treat refractory PJIs, yielding good clinical outcomes and no reinfection during follow-up [16,17,20]. CHA is an interesting carrier material because it functions as a bone scaffold and does not require secondary removal, unlike polymethylmethacrylate cement.

The current study has several limitations. First, it was a retrospective study with a small sample size. Randomized trials are necessary to definitively determine whether the use of CHA in implant retention procedures for PJI can improve infection-free survival. Second, the functional outcomes to evaluate the success of surgery were analyzed at different times. Additionally, the study cohort lacked a direct control group for comparison with or without the addition of antibiotic-impregnated CHA.

## 5. Conclusions

In conclusion, our findings suggest that antibiotic-impregnated CHA is highly effective in treating PJI after THA, even in cases of advanced disease, and results in satisfactory functional outcomes postoperatively.

## Figures and Tables

**Table 1:** Patient demographics for revision surgery with antibiotic-impregnated CHA treatment.

Male 7 Hips (6 Patients)	Female 6 Hips
Age (treatment of periprosthetic joint infection (PJI))	Average 61.4 years (35–71)
Osteoarthritis (OA)	6 hips
Rheumatoid arthritis (RA)	2 hips (1 patients)
Neck of femur fracture (FX)	5 hip
Follow up periods after treatment of PJI	Average 13.8 years (11~19.7 years)

**Table 2:** Demographics of patients who underwent revision surgery with antibiotic-impregnated CHA treatment.

Case	Sex	Age	Diagnoses	Previous Surgery	Onset Symptom	Organism	Age of THA (Months)	The Time From Surgery to Onset Symptom
1	F	62	OA	THA	Chill	MRSA	31	3 months
2	F	69	FX	Hemiarthroplasty	Hip pain	Stap. epidermidis	1	1 month
3	F	52	OA	THA	Hip pain	Stap. aureus	12	2 months
4	F	71	OA	THA	Fever, fistula	CNS	1	2 weeks
5	M	71	RA	THA [rt]	Hip pain	MSSA	43	2 weeks
5	M	71	RA	THA [lt]	Hip pain	MSSA	36	2 weeks
6	F	65	OA	THA	Chill, fistula	Streptococcus	15	11 months
7	F	67	FX	Hemiarthroplasty	Hip pain	Stap.capitis	6	1 month
8	M	66	FX	Hemiarthroplasty	Swelling	Candida glabrata /MRSA	24	7 months
9	M	58	FX	Hemiarthroplasty	Hip pain	MSSA	1	6 months
10	M	35	OA	THA	Hip pain	Stap. epidermidis	7	5 weeks
11	M	62	FX	THA	Hip pain	Unknown	22	2 months
12	M	60	OA	THA	Hip pain, swelling	Unknown	20	2 months

F, female; M, male; OA, osteoarthritis; RA, rheumatoid arthritis; Fx, neck of femur fracture. THA, total hip arthroplasty; Stap., staphylococcus; MRSA, methicillin-resistant staphylococcus aureus; MSSA, methicillin-susceptible staphylococcus aureus; CNS, coagulase-negative staphylococcus

**Table 3:** Clinical results of I&D and exchange with antibiotic-impregnated CHA for the treatment of PJI.

Case	Sex	Age	Antibiotics in the CHA Blocks (Numbers)	Antibiotics at Interim Period (Weeks)	Success/Failure	Treatment of Reinfection
1	F	62	VCM (2)	ABK, GM (9)	Success	
2	F	69	VCM, CTM (5)	FOMX, ABK, AMK, VCM, TEIC (8)	Failure (Reinfection)	2-stage revision
3	F	52	IPM/CS, CTM (4)	ABPC, CLDM, PIPC, IPM/CS (6)	Failure (Reinfection)	2-stage revision
4	F	71	VCM, AMK (3)	SBTPC, CTM, IPM/CS (8)	Success	
5	M	71	VCM, FOM (3)	CEZ, PIPC, TEIC, LVFX, CLDM, RFP (6)	Success	
5	M	71	VCM, FOM (3)	CEZ, PIPC, TEIC, LVFX, CLDM, RFP (6)	Success	
12	M	60	VCM (2)	CEZ (3)	Success	

F, female; M, male; I&D, irrigation and debridement. CTM, cefotiam; AMK, amikacin; FMOX, flomoxef; VCM, vancomycin; MINO, minocycline; IPM/CS, imipenem/cilastatin; PCG, benzylpenicillin; ABK, arbekacin; GM, gentamicin; TEIC, teicoplanin; ABPC, ampicillin; CLDM, clindamycin; PIPC, piperacillin; SBTPC, sultamicillin; CEZ, cefazolin; MEPM, meropenem; RFP, rifampicin; ABPC/SBT, ampicillin/sulbactam; LVFX, levofloxacin

**Table 4:** Clinical results of two-stage revision with antibiotic-impregnated CHA for the treatment of PJI.

Case	Sex	Age	Antibiotics in the CHA Blocks (Numbers)	Antibiotics at Interim Period (Weeks)	Interim Period (Weeks)	Antibiotics in the CHA Blocks (Numbers)	Antibiotics at Interim Period (Weeks)	Interim Period (Weeks)	Success /Failure
			Removed Implantation			Reimplantation (Two-Stage Exchange)			
2	F	69	VCM, CTM (20)	FMOX, ABK, GM, VCM, TEIC	8	VCM (5)	GM, AMK	2	Success
3	F	52	VCM, AMK, MINO (20)	ABPC, CLDM, PIPC, IPM/CS	6	FMOX, IPM/CS (5)	FMOX, IPM/CS	3	Success
6	F	65	CZOP, CTM, FMOX (36)	CZOP, CTM, FMOX	3	-	FMOX, CZP	2	Success
7	F	67	CTM, AMK (20)	GM, CMZ, ABK, VCM	4	-	FMOX, ABPC/SBT, AMK	2	Success
8	M	66	FOM, MCZ, TEIC	PIPC, MCZ	3 times of irrigation and debridement	TEIC, MCZ (2)	CEZ, ABK, TEIC, CFPN, CTRX, VCM, MEPM	10	Success
9	M	58	FOM, IPM/CS (30)	CTRX, CZP	3	IPM/CS (2)	CTRX, CZP	2	Success
10	M	35	VCM, AMK (30)	VCM, ABK	6	VCM (2)	VCM, ABK	2.5	Success
11	M	62	VCM, AMK (16)	CZP, PIPC	9	VCM (2)	CEZ	2	Success

F, female; M, male; I&D, irrigation and debridement. CTM, cefotiam; AMK, amikacin; FMOX, flomoxef; VCM, vancomycin; MINO, minocycline; IPM/CS, imipenem/cilastatin; PCG, benzylpenicillin; ABK, arbekacin; GM, gentamicin; TEIC, teicoplanin; ABPC, ampicillin; CLDM, clindamycin; PIPC, piperacillin; SBTPC, sultamicillin; CEZ, cefazolin; MEPM. Meropenem; RFP, rifampicin; ABPC/SBT, ampicillin/sulbactam; LVFX, levofloxacin.

**Table 5:** Functional outcomes and implant-related complications.

Case	Follow-Up	Follow up Periods After Treatment of PJI (Years)	Final JOA Score	Stress Shielding	Implant-Related Complications
1	Regularly visits	18.6	49	3	
2	Regularly visits	16.4	65	3	
3	Died of other causes	12.5	46	4	
4	Regularly visits	11.6	54	4	
5	Regularly visits	11	87	3	
5	Regularly visits	11	91	4	
6	Died of other causes	13.3	58	3	Cup revision due to aseptic cup loosening 10 years after surgery
7	Regularly visits	19.7	60	3	
8	Died of other causes	11.6	62	4	
9	Regularly visits	12.2	95	2	
10	Regularly visits	11.6	61	3	Dislocation 3 days after surgery, no redislocation
11	Regularly visits	12.4	85	3	
12	Regularly visits	13.4	88	3	

CHA: calcium hydroxyapatite, PJI: periprosthetic joint infection.

## Data Availability

The original contributions presented in this study are included in the article. Further inquiries can be directed to the corresponding author.
